# Supplementary material for: Nation-scale primary prevention to reduce newly incident adolescent drug use: the issue of lag time
Source: PeerJ. 2019 Feb 12;7:e6356. doi: 10.7717/peerj.6356 (PMC6376940; doi:10.7717/peerj.6356)
Supplement: Supplemental Information 1 [file peerj-07-6356-s001.docx]

| **Supplementary Table S1. Summary of main NSDUH methodological changes pertinent to the age of first alcoholic drink and first tobacco cigarette smoking.** | |
| --- | --- |
| **Year** | **General changes of assessment** |
| 1991 | - Sampling was redesigned to include Alaska and Hawaii, as were civilians living on military bases and individuals living in noninstitutional group quarters (such as homeless shelters, college dormitories, and boarding houses). |
| 1999 | - The assessment changed from paper-and-pencil to audio-computer assisted self-interview (ACASI) - Sample size increased to ~70,000 per year. |
| 2002 | - Respondents were provided with a monetary incentive. - The name of the survey changed from National Household Survey on Drug Abuse (NHSDA) to National Survey on Drug Use and Health (NSDUH). |
|  | **Questions about the age of first drink** |
| 1979 | **Please write in the age that shows about how old you were at the time you had your first drink.** |
| 1982 | **About how old were you the first time you had a glass of beer or a drink of liquor, such as whiskey, gin, scotch, etc.?** |
| 1985-1993 | **About how old were you the first time you had a glass of beer or wine or a drink of liquor, such as whiskey, gin, scotch, etc.? Do not include childhood sips that you might have had from an older person’s drink.** |
| 1994-1998 | The next few questions are about drinks of alcoholic beverages. By a “drink,” we mean a can or bottle of beer, a glass of wine or a wine cooler, a shot of liquor, or a mixed drink with liquor in it.  …  **How old were you the first time you had a drink of any alcoholic beverage? Do not include sips from another person’s drink.** |
| 1999-2015 | These questions are about drinks of alcoholic beverages. Throughout these questions, by a “drink,” we mean a can or bottle of beer, a glass of wine or a wine cooler, a shot of liquor, or a mixed drink with liquor in it. We are not asking about times when you only had a sip or two from a drink.  Examples of different types of alcoholic beverages.  **How old were you the first time you had a drink of an alcoholic beverage? Please do not include any time when you only had a sip or two from a drink.** |
|  | **Questions about the age of first cigarette smoke** |
| 1979-1993 | **About how old were you when you first tried a cigarette?** |
| 1994-1998 | **How old were you the first time you smoked a cigarette, even one or two puffs?** |
| 1999-2015 | **How old were you the first time you smoked part or all of a cigarette?** |

| **Supplementary Table S2. Sample Size Description for the ‘At-Risk’ Population for Newly Incident Alcohol Drinking Among 12-23 Year Olds. Data From the United States National Surveys on Drug Use and Health, 1979-2015 (n=358,205).** | | | | | | | | | | | |
| --- | --- | --- | --- | --- | --- | --- | --- | --- | --- | --- | --- |
| **Year** | **Age=12** | **Age=13** | **Age=14** | **Age=15** | **Age=16** | **Age=17** | **Age=18** | **Age=19** | **Age=20** | **Age=21** | **Age=22-23** |
| **1979** | 246 | 235 | 216 | 189 | 164 | 98 | 53 | 42 | 25 | 40 | 60 |
| **1982** | 212 | 205 | 155 | 152 | 102 | 61 | 22 | 18 | 14 | 11 | 39 |
| **1985** | 331 | 292 | 322 | 253 | 203 | 140 | 78 | 51 | 46 | 30 | 111 |
| **1988** | 428 | 439 | 427 | 385 | 289 | 202 | 84 | 44 | 41 | 29 | 79 |
| **1990** | 312 | 324 | 296 | 257 | 234 | 135 | 110 | 59 | 47 | 38 | 111 |
| **1991** | 1188 | 1263 | 1062 | 947 | 817 | 584 | 422 | 274 | 194 | 179 | 534 |
| **1992** | 1201 | 1151 | 1026 | 881 | 768 | 614 | 442 | 271 | 255 | 218 | 568 |
| **1993** | 1120 | 1134 | 1054 | 890 | 726 | 486 | 290 | 170 | 154 | 143 | 377 |
| **1994** | 727 | 775 | 703 | 607 | 491 | 305 | 200 | 123 | 84 | 96 | 258 |
| **1995** | 692 | 811 | 662 | 590 | 520 | 355 | 243 | 154 | 126 | 121 | 323 |
| **1996** | 673 | 731 | 709 | 598 | 520 | 388 | 294 | 174 | 148 | 151 | 380 |
| **1997** | 1235 | 1200 | 1206 | 1050 | 805 | 629 | 418 | 245 | 189 | 230 | 539 |
| **1998** | 1081 | 1080 | 1018 | 913 | 721 | 608 | 513 | 332 | 262 | 268 | 616 |
| **1999** | 2899 | 3056 | 2667 | 2228 | 1853 | 1331 | 1006 | 574 | 492 | 482 | 1063 |
| **2000** | 3083 | 3186 | 2847 | 2407 | 1910 | 1341 | 1069 | 592 | 476 | 560 | 1156 |
| **2001** | 2706 | 2892 | 2549 | 2084 | 1683 | 1189 | 968 | 566 | 475 | 497 | 1016 |
| **2002** | 3039 | 2788 | 2537 | 1955 | 1587 | 1158 | 850 | 531 | 385 | 458 | 857 |
| **2003** | 2835 | 2857 | 2502 | 2208 | 1739 | 1250 | 995 | 551 | 426 | 513 | 876 |
| **2004** | 2829 | 2964 | 2660 | 2220 | 1819 | 1297 | 1041 | 605 | 452 | 520 | 937 |
| **2005** | 2871 | 2883 | 2796 | 2356 | 1890 | 1395 | 1083 | 614 | 474 | 589 | 935 |
| **2006** | 2748 | 2866 | 2618 | 2361 | 1932 | 1459 | 1035 | 605 | 457 | 527 | 878 |
| **2007** | 2639 | 2727 | 2572 | 2389 | 1991 | 1423 | 1187 | 598 | 536 | 574 | 977 |
| **2008** | 2549 | 2631 | 2559 | 2439 | 2054 | 1597 | 1200 | 687 | 527 | 651 | 934 |
| **2009** | 2481 | 2645 | 2572 | 2485 | 2015 | 1633 | 1249 | 717 | 495 | 643 | 897 |
| **2010** | 2831 | 2957 | 2684 | 2496 | 2198 | 1763 | 1224 | 800 | 562 | 663 | 892 |
| **2011** | 2905 | 3124 | 2889 | 2705 | 2410 | 1775 | 1336 | 763 | 586 | 750 | 854 |
| **2012** | 2777 | 2684 | 2599 | 2520 | 2231 | 1652 | 1257 | 795 | 611 | 787 | 877 |
| **2013** | 2680 | 2856 | 2878 | 2628 | 2291 | 1632 | 1295 | 721 | 627 | 779 | 858 |
| **2014** | 2097 | 2214 | 2182 | 2052 | 1777 | 1249 | 999 | 576 | 456 | 612 | 645 |
| **2015** | 2038 | 2215 | 2227 | 2061 | 1775 | 1375 | 1151 | 643 | 527 | 673 | 791 |

| **Supplementary Table S3. Sample Size Description for the ‘At-Risk’ Population for Newly Incident Tobacco Cigarette Smoking Among 12-23 Year Olds. Data From the United States National Surveys on Drug Use and Health, 1979-2015 (n=429,348).** | | | | | | | | | | | |
| --- | --- | --- | --- | --- | --- | --- | --- | --- | --- | --- | --- |
| **Year** | **Age=12** | **Age=13** | **Age=14** | **Age=15** | **Age=16** | **Age=17** | **Age=18** | **Age=19** | **Age=20** | **Age=21** | **Age=22-23** |
| **1979** | 257 | 224 | 198 | 166 | 173 | 113 | 59 | 58 | 46 | 38 | 176 |
| **1982** | 211 | 193 | 170 | 144 | 114 | 90 | 40 | 28 | 34 | 45 | 152 |
| **1985** | 307 | 298 | 315 | 234 | 219 | 167 | 110 | 75 | 65 | 66 | 256 |
| **1988** | 397 | 425 | 399 | 352 | 307 | 294 | 95 | 67 | 52 | 59 | 202 |
| **1990** | 313 | 316 | 268 | 255 | 236 | 169 | 135 | 101 | 85 | 73 | 295 |
| **1991** | 1135 | 1240 | 1056 | 942 | 923 | 729 | 578 | 455 | 427 | 355 | 1321 |
| **1992** | 1185 | 1081 | 984 | 863 | 844 | 736 | 552 | 431 | 388 | 366 | 1397 |
| **1993** | 1110 | 1095 | 1016 | 891 | 756 | 657 | 351 | 263 | 266 | 222 | 804 |
| **1994** | 729 | 756 | 696 | 605 | 485 | 381 | 225 | 157 | 122 | 140 | 488 |
| **1995** | 706 | 783 | 646 | 579 | 524 | 394 | 269 | 198 | 187 | 165 | 654 |
| **1996** | 666 | 709 | 689 | 570 | 524 | 423 | 320 | 250 | 227 | 204 | 769 |
| **1997** | 1206 | 1160 | 1158 | 955 | 808 | 656 | 449 | 329 | 293 | 275 | 1085 |
| **1998** | 1075 | 1057 | 956 | 858 | 674 | 612 | 529 | 416 | 355 | 320 | 1260 |
| **1999** | 2877 | 3021 | 2562 | 2057 | 1829 | 1414 | 1120 | 766 | 707 | 648 | 2472 |
| **2000** | 3016 | 3179 | 2781 | 2306 | 1873 | 1463 | 1131 | 848 | 749 | 690 | 2743 |
| **2001** | 2725 | 2786 | 2478 | 2063 | 1754 | 1374 | 1075 | 824 | 668 | 649 | 2539 |
| **2002** | 2997 | 2825 | 2467 | 1963 | 1678 | 1333 | 1030 | 799 | 667 | 604 | 2199 |
| **2003** | 2873 | 2853 | 2519 | 2237 | 1904 | 1511 | 1209 | 846 | 656 | 673 | 2353 |
| **2004** | 2816 | 2962 | 2655 | 2350 | 2040 | 1667 | 1351 | 966 | 756 | 671 | 2411 |
| **2005** | 2889 | 2887 | 2781 | 2559 | 2239 | 1771 | 1358 | 978 | 795 | 768 | 2552 |
| **2006** | 2782 | 2852 | 2764 | 2537 | 2285 | 1855 | 1439 | 996 | 814 | 748 | 2471 |
| **2007** | 2676 | 2770 | 2584 | 2571 | 2252 | 1946 | 1588 | 1063 | 887 | 818 | 2606 |
| **2008** | 2564 | 2658 | 2654 | 2614 | 2375 | 2069 | 1606 | 1140 | 905 | 860 | 2774 |
| **2009** | 2459 | 2669 | 2687 | 2651 | 2398 | 2135 | 1667 | 1219 | 963 | 868 | 2835 |
| **2010** | 2831 | 2935 | 2780 | 2697 | 2531 | 2243 | 1664 | 1224 | 1052 | 882 | 2969 |
| **2011** | 2908 | 3135 | 2958 | 2884 | 2733 | 2367 | 1738 | 1206 | 1082 | 913 | 3073 |
| **2012** | 2781 | 2707 | 2635 | 2639 | 2512 | 2252 | 1610 | 1235 | 1064 | 976 | 3200 |
| **2013** | 2721 | 2883 | 2859 | 2709 | 2608 | 2237 | 1682 | 1181 | 1078 | 937 | 3274 |
| **2014** | 2084 | 2207 | 2226 | 2148 | 1992 | 1681 | 1271 | 910 | 796 | 773 | 2396 |
| **2015** | 2038 | 2197 | 2277 | 2188 | 1978 | 1786 | 1452 | 1037 | 955 | 844 | 2967 |

| **Supplement Table S3. Estimated Annual Incidence (% and 95% confidence intervals) of Alcohol Drinking in the United States Among 12-23 Year Olds From 1979 to 2015. Data From the United States National Surveys on Drug Use and Health, 1979-2015 (n=358,205).** | | | | | | | | | | | |
| --- | --- | --- | --- | --- | --- | --- | --- | --- | --- | --- | --- |
| **Year** | **Age=12** | **Age=13** | **Age=14** | **Age=15** | **Age=16** | **Age=17** | **Age=18** | **Age=19** | **Age=20** | **Age=21** | **Age=22-23** |
| **1979** | 31 (24,40) | 36 (29,44) | 43 (34,52) | 60 (48,70) | 59 (48,69) | 64 (52,75) | 73 (59,83) | 59 (46,71) | 50 (24,75) | 59 (42,75) | 24 (13,39) |
| **1982** | 22 (16,30) | 37 (28,47) | 32 (23,42) | 49 (40,58) | 50 (40,59) | 44 (27,63) | 38 (18,63) | 33 (15,58) | 34 (15,61) | 11 (2,45) | 7 (2,25) |
| **1985** | 16 (11,23) | 28 (23,33) | 30 (24,38) | 48 (39,56) | 46 (37,55) | 44 (33,56) | 38 (25,53) | 44 (28,62) | 38 (22,58) | 34 (16,58) | 6 (2,18) |
| **1988** | 14 (10,20) | 23 (18,28) | 26 (21,32) | 37 (31,43) | 44 (38,51) | 36 (25,48) | 35 (22,52) | 26 (12,47) | 27 (13,48) | 41 (18,69) | 5 (2,10) |
| **1990** | 11 (8,16) | 21 (15,29) | 29 (22,36) | 35 (26,45) | 36 (27,46) | 38 (27,50) | 34 (25,44) | 26 (14,42) | 20 (7,44) | 33 (16,55) | 8 (4,18) |
| **1991** | 15 (12,18) | 20 (16,25) | 27 (23,31) | 26 (22,30) | 35 (31,40) | 37 (30,45) | 43 (34,52) | 36 (27,47) | 23 (14,35) | 29 (19,40) | 5 (3,8) |
| **1992** | 8 (6,11) | 14 (11,18) | 20 (17,24) | 28 (23,33) | 37 (31,42) | 33 (26,40) | 30 (22,39) | 24 (16,36) | 17 (11,25) | 34 (23,48) | 4 (2,8) |
| **1993** | 9 (6,12) | 17 (13,22) | 23 (19,28) | 30 (25,36) | 32 (26,38) | 39 (31,48) | 43 (34,52) | 21 (13,33) | 15 (8,25) | 39 (26,54) | 6 (3,13) |
| **1994** | 11 (8,13) | 18 (14,21) | 23 (19,27) | 33 (29,38) | 39 (34,45) | 34 (26,43) | 42 (32,52) | 21 (13,33) | 20 (11,35) | 40 (28,54) | 4 (2,8) |
| **1995** | 9 (7,12) | 18 (15,21) | 28 (25,32) | 31 (26,36) | 34 (29,40) | 33 (26,41) | 33 (26,42) | 28 (19,38) | 29 (20,41) | 44 (33,56) | 4 (2,8) |
| **1996** | 8 (6,11) | 17 (14,21) | 23 (19,27) | 32 (27,37) | 35 (29,40) | 35 (29,42) | 28 (21,37) | 21 (14,32) | 26 (17,38) | 44 (33,56) | 5 (3,9) |
| **1997** | 12 (9,15) | 20 (17,25) | 26 (23,29) | 30 (26,34) | 34 (30,39) | 34 (28,40) | 37 (31,45) | 37 (28,47) | 34 (24,46) | 48 (36,62) | 5 (3,8) |
| **1998** | 7 (5,10) | 15 (12,19) | 24 (21,28) | 30 (26,35) | 36 (30,43) | 36 (30,42) | 40 (33,48) | 25 (17,36) | 28 (20,37) | 44 (35,54) | 5 (3,8) |
| **1999** | 12 (11,13) | 18 (17,20) | 26 (24,28) | 34 (32,36) | 34 (32,36) | 34 (30,37) | 36 (33,40) | 28 (23,33) | 23 (19,29) | 44 (39,50) | 6 (5,9) |
| **2000** | 12 (10,13) | 18 (17,20) | 24 (23,26) | 32 (30,34) | 33 (31,36) | 35 (32,38) | 38 (35,41) | 30 (26,36) | 25 (21,29) | 47 (43,51) | 7 (5,10) |
| **2001** | 10 (9,12) | 19 (17,20) | 26 (24,28) | 35 (32,38) | 34 (32,37) | 36 (33,39) | 37 (34,41) | 26 (22,30) | 23 (19,28) | 53 (46,59) | 7 (5,9) |
| **2002** | 10 (8,11) | 16 (15,18) | 25 (24,27) | 34 (31,37) | 36 (34,39) | 41 (37,45) | 39 (35,43) | 27 (23,32) | 28 (23,34) | 57 (52,62) | 8 (6,11) |
| **2003** | 9 (8,10) | 17 (15,19) | 25 (22,27) | 36 (33,38) | 36 (33,39) | 36 (32,40) | 41 (37,45) | 30 (26,35) | 37 (30,45) | 56 (51,61) | 9 (6,12) |
| **2004** | 9 (8,11) | 16 (14,18) | 26 (24,29) | 32 (30,34) | 37 (35,40) | 40 (35,44) | 39 (35,43) | 28 (24,33) | 27 (22,34) | 57 (52,62) | 8 (6,11) |
| **2005** | 9 (8,10) | 15 (13,17) | 26 (24,28) | 33 (31,36) | 37 (34,39) | 37 (34,40) | 41 (36,45) | 32 (28,37) | 24 (19,29) | 55 (49,61) | 6 (5,9) |
| **2006** | 9 (7,10) | 17 (15,19) | 25 (22,27) | 32 (30,34) | 34 (32,37) | 38 (35,41) | 43 (39,47) | 32 (28,37) | 31 (25,37) | 54 (48,59) | 7 (5,11) |
| **2007** | 8 (6,9) | 14 (12,16) | 25 (23,27) | 32 (29,35) | 35 (32,38) | 36 (32,40) | 43 (39,47) | 27 (22,32) | 29 (24,34) | 62 (56,67) | 7 (5,9) |
| **2008** | 8 (7,9) | 14 (12,16) | 24 (22,26) | 32 (30,35) | 35 (31,38) | 36 (33,39) | 41 (37,44) | 31 (26,35) | 24 (20,29) | 60 (54,64) | 10 (8,14) |
| **2009** | 8 (7,10) | 15 (13,17) | 22 (20,25) | 30 (28,32) | 37 (34,40) | 36 (33,39) | 43 (40,46) | 29 (25,33) | 30 (25,35) | 63 (59,67) | 9 (7,11) |
| **2010** | 7 (5,8) | 13 (12,15) | 23 (21,26) | 30 (28,33) | 33 (30,36) | 34 (31,37) | 42 (38,46) | 32 (28,37) | 31 (27,36) | 67 (63,72) | 7 (6,9) |
| **2011** | 7 (6,9) | 13 (12,15) | 20 (18,22) | 29 (28,31) | 33 (30,36) | 33 (29,36) | 38 (34,41) | 23 (19,28) | 23 (19,28) | 66 (61,71) | 11 (7,16) |
| **2012** | 6 (5,7) | 12 (10,14) | 20 (18,22) | 27 (25,30) | 31 (29,33) | 30 (27,34) | 40 (36,44) | 27 (23,32) | 28 (24,32) | 61 (56,66) | 12 (9,15) |
| **2013** | 4 (3,5) | 10 (8,12) | 17 (15,18) | 27 (25,29) | 31 (29,34) | 34 (31,37) | 37 (34,41) | 26 (22,31) | 24 (20,29) | 69 (64,74) | 10 (8,12) |
| **2014** | 5 (4,6) | 10 (9,12) | 15 (14,17) | 25 (23,28) | 30 (28,33) | 35 (31,39) | 39 (35,44) | 26 (22,31) | 29 (25,35) | 69 (63,73) | 8 (5,11) |
| **2015** | 4 (3,6) | 10 (9,12) | 17 (15,19) | 25 (22,27) | 31 (28,34) | 31 (27,34) | 37 (33,41) | 25 (21,29) | 27 (23,31) | 70 (65,74) | 9 (7,12) |

| **Supplement Table S4. Estimated Annual Incidence (% and 95% confidence intervals) of Tobacco Cigarette Smoking in the United States Among 12-23 Year Olds From 1979 to 2015. Data From the United States National Surveys on Drug Use and Health, 1979-2015 (n=429,348).** | | | | | | | | | | | |
| --- | --- | --- | --- | --- | --- | --- | --- | --- | --- | --- | --- |
| **Year** | **Age=12** | **Age=13** | **Age=14** | **Age=15** | **Age=16** | **Age=17** | **Age=18** | **Age=19** | **Age=20** | **Age=21** | **Age=22-23** |
| **1979** | 16 (11,23) | 20 (14,27) | 18 (12,26) | 13 (7,23) | 15 (10,21) | 17 (11,25) | 10 (5,20) | 7 (3,18) | 11 (3,32) | 7 (2,19) | 1 (0,8) |
| **1982** | 17 (12,23) | 12 (7,21) | 16 (9,26) | 20 (13,30) | 11 (5,22) | 12 (5,27) | 5 (1,17) | 6 (1,23) | 7 (2,21) | 4 (1,15) | 3 (1,8) |
| **1985** | 9 (6,15) | 19 (14,26) | 16 (11,21) | 20 (14,28) | 12 (8,17) | 12 (6,22) | 6 (3,11) | 9 (3,25) | 4 (1,11) | 1 (0,5) | 1 (0,4) |
| **1988** | 12 (8,17) | 13 (10,18) | 19 (14,25) | 13 (9,19) | 14 (9,20) | 17 (10,30) | 10 (5,21) | 9 (3,23) | 5 (1,16) | 0 (.,.) | 2 (1,6) |
| **1990** | 10 (6,17) | 10 (6,16) | 12 (7,19) | 16 (11,24) | 14 (9,21) | 14 (7,27) | 9 (4,19) | 10 (4,23) | 5 (2,15) | 4 (1,17) | 1 (0,4) |
| **1991** | 10 (8,12) | 13 (10,16) | 9 (7,12) | 15 (12,19) | 15 (11,19) | 16 (11,22) | 14 (9,21) | 9 (5,15) | 9 (5,16) | 7 (3,15) | 1 (0,1) |
| **1992** | 9 (6,12) | 10 (8,14) | 11 (8,14) | 13 (10,17) | 13 (10,18) | 11 (7,16) | 12 (8,18) | 8 (4,15) | 7 (4,14) | 11 (6,20) | 1 (1,2) |
| **1993** | 9 (6,13) | 8 (6,11) | 14 (10,19) | 19 (15,24) | 14 (9,20) | 17 (11,24) | 18 (11,26) | 7 (3,14) | 6 (3,13) | 5 (2,12) | 1 (0,2) |
| **1994** | 14 (11,18) | 15 (12,18) | 16 (13,20) | 23 (19,27) | 21 (18,26) | 18 (12,25) | 21 (15,29) | 10 (5,20) | 11 (6,19) | 12 (7,20) | 2 (1,5) |
| **1995** | 12 (9,15) | 18 (15,21) | 24 (19,28) | 22 (18,28) | 19 (14,25) | 25 (21,31) | 16 (11,22) | 11 (6,18) | 14 (8,24) | 9 (4,19) | 1 (0,2) |
| **1996** | 12 (8,17) | 15 (12,19) | 18 (15,21) | 21 (17,26) | 22 (17,27) | 20 (16,25) | 15 (12,19) | 10 (5,17) | 10 (5,17) | 7 (4,12) | 3 (2,5) |
| **1997** | 11 (8,13) | 19 (15,23) | 20 (18,24) | 19 (16,23) | 23 (18,28) | 22 (15,30) | 21 (15,27) | 16 (11,22) | 9 (6,15) | 9 (5,15) | 2 (1,4) |
| **1998** | 11 (8,14) | 15 (12,19) | 15 (12,20) | 18 (15,22) | 18 (14,23) | 20 (15,26) | 17 (12,24) | 14 (9,21) | 9 (6,13) | 13 (8,20) | 3 (2,6) |
| **1999** | 10 (9,11) | 15 (13,16) | 16 (14,18) | 16 (14,18) | 19 (17,21) | 18 (16,21) | 18 (15,21) | 14 (11,18) | 10 (7,12) | 7 (5,9) | 2 (1,2) |
| **2000** | 7 (6,8) | 11 (10,13) | 14 (13,16) | 19 (17,20) | 16 (14,19) | 17 (14,20) | 14 (12,17) | 12 (9,15) | 9 (7,12) | 8 (6,10) | 2 (1,3) |
| **2001** | 8 (7,9) | 11 (10,13) | 13 (11,15) | 17 (15,19) | 16 (14,18) | 14 (12,17) | 16 (13,19) | 10 (8,13) | 9 (7,12) | 8 (6,11) | 2 (1,3) |
| **2002** | 6 (5,7) | 11 (9,12) | 12 (10,13) | 15 (13,18) | 16 (14,19) | 16 (14,19) | 19 (16,23) | 9 (7,11) | 9 (6,12) | 7 (5,10) | 2 (1,3) |
| **2003** | 5 (4,6) | 8 (7,9) | 12 (11,14) | 15 (13,17) | 17 (15,20) | 14 (12,17) | 17 (15,20) | 11 (8,14) | 6 (4,8) | 10 (7,14) | 2 (1,3) |
| **2004** | 6 (5,7) | 9 (7,10) | 13 (11,14) | 14 (12,15) | 18 (16,21) | 18 (15,21) | 20 (18,23) | 13 (10,16) | 9 (7,12) | 6 (4,8) | 3 (2,4) |
| **2005** | 6 (5,8) | 8 (7,9) | 11 (10,13) | 15 (13,16) | 17 (15,19) | 17 (15,20) | 19 (16,22) | 12 (9,16) | 10 (7,13) | 7 (5,10) | 2 (1,3) |
| **2006** | 6 (4,7) | 7 (6,8) | 12 (10,13) | 14 (13,16) | 15 (14,17) | 16 (14,18) | 23 (20,27) | 13 (11,15) | 11 (9,14) | 7 (5,9) | 2 (2,3) |
| **2007** | 4 (3,5) | 7 (6,8) | 10 (8,11) | 13 (11,15) | 14 (12,16) | 17 (15,20) | 23 (20,26) | 15 (12,18) | 9 (6,12) | 7 (5,10) | 3 (2,4) |
| **2008** | 3 (3,5) | 7 (5,9) | 11 (9,13) | 14 (12,16) | 15 (14,17) | 17 (15,20) | 23 (20,26) | 14 (11,17) | 8 (6,10) | 8 (6,11) | 2 (2,3) |
| **2009** | 4 (3,5) | 7 (5,8) | 10 (9,12) | 14 (12,16) | 16 (14,18) | 18 (16,20) | 23 (20,26) | 13 (11,16) | 10 (8,14) | 8 (5,12) | 2 (2,3) |
| **2010** | 4 (3,6) | 5 (4,6) | 11 (10,12) | 14 (12,15) | 14 (12,17) | 16 (15,18) | 22 (19,25) | 15 (12,17) | 11 (8,14) | 6 (4,8) | 3 (2,4) |
| **2011** | 3 (2,4) | 5 (4,6) | 9 (7,10) | 13 (11,14) | 14 (12,16) | 15 (13,18) | 23 (21,26) | 13 (10,17) | 10 (8,13) | 7 (5,10) | 2 (2,3) |
| **2012** | 2 (2,3) | 5 (4,6) | 8 (6,9) | 12 (10,14) | 12 (10,13) | 16 (13,19) | 22 (19,24) | 14 (11,17) | 11 (8,14) | 9 (6,12) | 2 (2,3) |
| **2013** | 2 (1,3) | 5 (4,6) | 6 (5,7) | 10 (8,12) | 12 (10,14) | 14 (12,16) | 19 (17,22) | 13 (10,17) | 9 (6,12) | 7 (5,10) | 2 (1,3) |
| **2014** | 2 (2,3) | 4 (3,5) | 5 (4,6) | 8 (7,9) | 11 (10,13) | 13 (11,15) | 19 (16,22) | 12 (9,15) | 12 (8,16) | 8 (6,9) | 2 (2,3) |
| **2015** | 2 (2,3) | 3 (3,5) | 6 (5,7) | 8 (7,10) | 10 (9,12) | 11 (9,13) | 19 (16,22) | 10 (8,13) | 10 (8,13) | 6 (5,9) | 2 (2,3) |

| **Supplementary Figure 1. Estimated Annual Incidence (%) of Alcohol Drinking in the United States Among 12-23 Year Olds From 1979 to 2015. Data From the United States National Surveys on Drug Use and Health, 1979-2015 (n=358,205).** |
| --- |
|  |
|  |
|  |
|  |
|  |

| **Supplementary Figure 2. Estimated Annual Incidence (%) of Tobacco Cigarette Smoking in the United States Among 12-23 Year Olds From 1979 to 2015. Data From the United States National Surveys on Drug Use and Health, 1979-2015 (n=429,348).** |
| --- |
|  |
|  |
|  |
|  |
|  |
